# Decreased IgG Antibody Response to Viral Protein Mimotopes of Oncogenic Merkel Cell Polyomavirus in Sera From Healthy Elderly Subjects

**DOI:** 10.3389/fimmu.2021.738486

**Published:** 2021-10-18

**Authors:** Chiara Mazziotta, Carmen Lanzillotti, Marcello Govoni, Giulia Pellielo, Elisa Mazzoni, Mauro Tognon, Fernanda Martini, John Charles Rotondo

**Affiliations:** ^1^ Department of Medical Sciences, University of Ferrara, Ferrara, Italy; ^2^ Center for Studies on Gender Medicine, Department of Medical Sciences, University of Ferrara, Ferrara, Italy; ^3^ Laboratory for Technologies of Advanced Therapies (LTTA), University of Ferrara, Ferrara, Italy

**Keywords:** elderly individuals, Merkel cell polyomavirus, MCPyV, Merkel cell carcinoma, immunological assay, enzyme-linked immunosorbent assay, immune system, immunosenescence

## Abstract

Merkel cell polyomavirus (MCPyV) is the main causative agent of Merkel cell carcinoma (MCC), a rare but aggressive skin tumor with a typical presentation age >60 years. MCPyV is ubiquitous in humans. After an early-age primary infection, MCPyV establishes a clinically asymptomatic lifelong infection. In immunocompromised patients/individuals, including elders, MCC can arise following an increase in MCPyV replication events. Elders are prone to develop immunesenescence and therefore represent an important group to investigate. In addition, detailed information on MCPyV serology in elders has been debated. These findings cumulatively indicate the need for new research verifying the impact of MCPyV infection in elderly subjects (ES). Herein, sera from 226 ES, aged 66–100 years, were analyzed for anti-MCPyV IgGs with an indirect ELISA using peptides mimicking epitopes from the MCPyV capsid proteins VP1-2. Immunological data from sera belonging to a cohort of healthy subjects (HS) (n = 548) aged 18–65 years, reported in our previous study, were also included for comparisons. Age-/gender-specific seroprevalence and serological profiles were investigated. MCPyV seroprevalence in ES was 63.7% (144/226). Age-specific MCPyV seroprevalence resulted as 62.5% (25/40), 71.7% (33/46), 64.9% (37/57), 63.8% (30/47), and 52.8% (19/36) in ES aged 66–70, 71–75, 76–80, 81–85, and 86–100 years, respectively (p > 0.05). MCPyV seroprevalence was 67% (71/106) and 61% (73/120) in ES males and females, respectively (p > 0.05). Lack of age-/gender-related variations in terms of MCPyV serological profiles was found in ES (p > 0.05). Notably, serological profile analyses indicated lower optical densities (ODs) in ES compared with HS (p < 0.05), while lower ODs were also determined in ES males compared with HS males (p < 0.05). Our data cumulatively suggest that oncogenic MCPyV circulates in elders asymptomatically at a relatively high prevalence, while immunesenescence might be responsible for a decreased IgG antibody response to MCPyV, thereby potentially leading to an increase in MCPyV replication levels. In the worse scenario, alongside other factors, MCPyV might drive MCC carcinogenesis, as described in elders with over 60 years of age.

## Introduction

Merkel cell polyomavirus (MCPyV) is a small DNA virus with oncogenic potential (1). MCPyV is the main causative factor of Merkel cell carcinoma (MCC), a rare but aggressive skin tumor with a presentation age above 60 years (2). DNA sequences belonging to this polyomavirus (PyV) have been reported in ~80% of MCC tissues (1).

MCPyV genome is made up of a circular double-stranded DNA of ~5,400 base pairs containing three functional domains (3). These domains encompass a non-coding control region and early and late coding regions (4, 5), which regulate the early and late gene expression, respectively (6). The early region is involved in MCPyV replication initiation, whereas it comprises genes encoding for transcripts generated by alternative splicing, including the two proteins with oncogenic potential named large T (LT) and small (sT) antigens. The continuous expression of both LT and sT, alongside viral DNA integration into host genome, is required for the MCC onset and development (7–9). Additionally, LT/sT expression also appears to be linked to improper DNA methylation (10), an epigenetic process regulating gene expression (11). The late region encodes for MCPyV capsid proteins, known as major capsid protein 1 (VP1) and minor capsid protein 2 (VP2) (7). Differently from other PyVs, MCPyV does not express the VP3 protein (4).

Previous serological studies aimed to detect antibodies against MCPyV VPs have reported conflicting data on MCPyV prevalence in adulthood and middle/advanced age, with a seroprevalence ranging between 46% and 87% being described in healthy adults (3, 12–26) and between 58% and 95% in the elderly (12–15, 21–23, 25–29). Despite these conflicting rates, previous studies concordantly imply that MCPyV appears to be almost ubiquitous in the general population, which is asymptomatically infected (18, 27, 30, 31). After primary infection, which occurs during childhood (28, 32, 33), MCPyV establishes a lifelong but inoffensive infection in healthy individuals (32, 34). However, in certain circumstances, such as immune system impairment in the host, increased viral activity can occur. MCPyV may induce the dysregulated expression of host cell genes, which ultimately increase the MCC occurrence. Immunosuppression plays an important role in MCC onset (9, 35–37). Indeed, a higher occurrence of MCC has been observed in patients with immunocompromising conditions, including human immunodeficiency virus (HIV) infection (38–41), leukemia, and other hematologic malignancies (42, 43), as well as patients being pharmacologically treated for autoimmune diseases, solid organ transplantation, and cancers of other types (7, 9, 44–46). Notably, as only 10% of MCC patients have overt immunodeficiency, it is likely that in most patients cancer development is promoted by a loss of antiviral/cancer immune surveillance during advanced age (38, 47). Physiological immune impairment as a result of aging, or immunosenescence, could therefore favor high levels of MCPyV replication, ultimately leading to MCC onset. It has been reported that MCPyV viral load increases among elders, suggesting an age-related association of viral replication in the host (34, 48). Considering these aspects, assessing the impact of MCPyV infection in various immunocompromised groups, including elders, can improve early identification of individuals at risk of developing MCC.

In a previous study, we developed and validated a specific and sensitive indirect ELISA method with two linear synthetic peptides/mimotopes that mimic the MCPyV VP1 and VP2 antigens to detect circulating IgGs against MCPyV immunogenic antigens (18). Herein, we aimed to evaluate the impact of MCPyV infection in elders by taking advantage from our recently developed immunoassay. Specifically, the prevalence of serum IgGs against MCPyV in healthy elderly subjects (ES) aged from 66 to 100 years was investigated. MCPyV seroprevalence and serological profiles were therefore determined in age- and gender-stratified ES. It is worth noting that MCPyV infection in ES is of paramount importance for identifying elderly individuals potentially at risk of developing MCC in healthy populations.

## Materials and Methods

### Human Sera

Serum samples were obtained from n = 226 (mean age ± standard deviation of mean [SD], 78 ± [7] years) healthy ES. Samples were stored at −80°C until testing. Analyses were conducted in age-stratified ES, i.e., 66–70 (n = 40), 71–75 (n = 46), 76–80 (n = 57), 81–85 (n = 47), and 86–100 (n = 36) year-old groups. In addition, data from a set of sera belonging to a cohort of healthy subjects (HS) (n = 548, mean age ± SD, 42 ± [13] years) aged 18–65 years, reported in our previous study (18), were included for comparisons. Specifically, HS were stratified herein according to age in 18–25 (n = 78), 26–30 (n = 52), 31–35 (n = 59), 36–40 (n = 61), 41–45 (n = 76), 46–50 (n = 65), 51–55 (n = 50), 56–60 (n = 55), and 61–65 (n = 52) year-old groups. Serum samples were collected at the Clinical Laboratory Analysis, University Hospital of Ferrara, Ferrara, Italy, from discarded laboratory analysis samples, after routine analyses, before their destruction by incineration. The hospital records indicated these samples as belonging to healthy subjects. Indeed, blood analysis parameters were all in the normal index range. In addition, sera were collected anonymously and coded with indications of age and gender. Written informed consent was obtained from all subjects. The County Ethical Committee, Ferrara, Italy, approved the study (ID: 151078).

### Merkel Cell Polyomavirus VP1 and VP2 Linear Synthetic Peptides

A recently developed and validated indirect ELISA was employed to detect specific IgGs against MCPyV in sera from ES, as described (18). The indirect ELISA provides the use of two linear synthetic peptides/mimotopes, known as MCPyV VP1 S and VP2 F (S and F peptides), which have previously been designed and employed as mimotopes for detecting circulating IgGs against MCPyV in a group of HS (18). The synthetic peptides were synthesized using standard procedures and purchased from UFPeptides s.r.l., Ferrara, Italy.

Amino acid (a.a.) sequences of VP1 S (24 a.a. residues) and VP2 F (25 a.a. residues) peptides, are as follows:VP1 S: NH_2_-NSPDLPTTSNWYTYTYDLQPKGSS-COOH,VP2 F: NH_2_-SLSPTSRLQIQSNLVNLILNSRWVF-COOH.

### Indirect ELISA

Indirect ELISA was performed as reported (18). Immunological plates (Nunc-immuno plate PolySorp, Thermo Fisher Scientific, Milan, Italy) were coated with 5 μg of S and/or F peptide for each well and diluted in 100 μl of Coating Buffer 1×, pH 9.6 (Candor Bioscience, Wangen, Germany). The plates coated with peptides were incubated at 4°C for 16 h. Subsequently, immunological plates were washed three times with Washing Buffer (Candor Bioscience, Wangen, Germany), to remove the unbound peptides. During the blocking phase, 200 μl/well of blocking solution containing the casein and Tween detergent (Candor Bioscience, Wangen, Germany) was added to each well and incubated at 37°C for 90 min. Plates were rinsed three times with Washing Buffer, before adding serum samples. Each well was covered with 100 μl containing the serum samples diluted 1:20 in low cross-buffer (Candor Bioscience, Wangen, Germany). Sera in each plate comprise the following: i) positive controls, represented by immune human sera derived from patients with MCPyV-positive MCC (18); ii) negative controls, represented by three human MCPyV-negative sera (18); and iii) sera from ES under analysis. Immunological plates with sera were incubated at 37°C for 90 min. Each sample was analyzed in three replicates. Wells were washed three times, and the secondary antibody was added to each sample. This solution consists of a goat anti-human IgG heavy (H) and light (L) chain specific peroxidase conjugate (Calbiochem-Merck, Darmstadt, Germany) diluted 1:10,000 in low cross-buffer. The solution was added to each well, and plates were incubated at room temperature (RT) for 90 min. After 90 min of incubation, plates were washed three times, and then 100 μl of 2,2′-azino-bis-3-ethylbenzthiazoline-6-sulfonic acid (ABTS) solution (Sigma-Aldrich, Milan, Italy) was added to each well. Plates were incubated at RT for 45 min. ABTS reacted with the peroxidase enzyme to yield the color reaction. Finally, the plate was read spectrophotometrically (Thermo Electron Corp., model Multiskan EX, Vantaa, Finland) at a wavelength (λ) of 405 nm. Color intensity in wells was determined by optical density (OD) reading. The OD readings correspond to the amount of immune complexes formed by the specific antibodies bound to S and F synthetic peptides/mimotopes.

The cutoff for each S and F synthetic peptide was determined in each indirect ELISA run, as the mean of the OD readings of n = 3 negative control sera plus three standard deviations of mean (mean +3 SDs) (18, 49, 50), as described previously for other ELISA methods (51, 52). Immune serum samples were considered MCPyV-positive when reacting to both S and F synthetic peptides, in three replica ELISA experiments carried out by three independent operators, without data variability.

Indirect ELISA dilutional linearity (accuracy) was assessed by testing n = 15 of 10-fold diluted samples, from 1:20 to 1:2,560 (53). Sera with high (n = 5), medium (n = 5), and low (n = 5) OD values were selected and tested in triplicate (18).

### Statistical Analysis

MCPyV seroprevalence rates were statistically analyzed applying the two-sided chi-square test for comparing ES and HS groups as well as age-/gender-stratified ES and HS cohorts (54, 55). OD values from the same ES and HS groups/cohorts were analyzed with the D’Agostino–Pearson normality test, and then parametric and non-parametric tests were applied according to normal and non-normal variables, respectively, as reported (53, 56). In detail, ODs from age-stratified ES and HS cohorts were analyzed with one-way ANOVA and Kruskal–Wallis multiple comparison tests, according to normal and non-normal variables, respectively (OD medians, 95% CI). ODs from the entire ES and HS groups as well as gender-stratified ES and HS cohorts were analyzed with Mann–Whitney U test. Spearman’s correlation coefficient r was used to evaluate the OD concordance between S and F peptides. Linear regression R^2^ coefficient was used to evaluate the agreement between sample dilutions and ODs, for both S and F peptides. Statistical analyses were carried out using Graph Pad Prism version 8.0 for Windows (Graph Pad, La Jolla, USA) (57). p-Values <0.05 were considered statistically significant (58).

## Results

### Indirect ELISA Reliability Assessment

**Figure 1:**
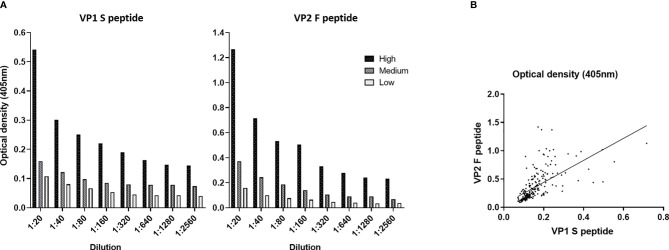
Dilution linearity and correlation of optical density (OD) values obtained using Merkel cell polyomavirus (MCPyV) VP1 S and VP2 F peptides. **(A)** OD response to serial dilutions (1:20, 1:80, 1:160, 1:320, 1:640, 1:1,280, and 1:2,560) was evaluated on n = 15 MCPyV-seropositive sera with known high (n = 5), medium (n = 5), and low (n = 5) ODs. Each dilution was assayed in triplicate for each MCPyV VP1 S and VP2 F peptide; and OD values and sera dilutions were compared by linear regression analysis. High correlation between ODs and dilutions was found for VP1 S peptide with an R^2^ of 0.9786 (p < 0.0001), 0.9959 (p < 0.0001), and 0.9768 (p < 0.0001) for samples with high, medium, and low ODs, respectively. Good correlation between ODs and dilutions was found when VP2 F peptide was employed, with an R^2^ of 0.9773 (p < 0.0001) for sample with known high ODs, 0.9784 (p < 0.0001) for samples with medium ODs, and 0.9846 (p < 0.0001) for samples with low ODs. **(B)** The concordance in ODs between the VP1 S and VP2 F peptides was evaluated on the entire set of sera from elderly subjects (ES) (n = 226) using Spearman’s correlation analysis. A good correlation between S peptide and F peptide was found, with an r of 0.7991 and a p < 0.0001.

In the first phase of our investigation, the accuracy of the indirect ELISA was determined by performing serial dilutions, from 1:20 to 1:2,560, on three sets of MCPyV-seropositive samples (n = 15) from our previous study (18), with known high (n = 5), medium (n = 5), and low (n = 5) OD values. For each peptide, dilutions were assayed in triplicate. Subsequently, ODs and dilution values were compared. The assay had a high correlation between ODs and sample dilutions when the S peptide was employed, with an R^2^ of 0.9786 (p < 0.0001), 0.9959 (p < 0.0001), and 0.9768 (p < 0.0001) for samples with high, medium, and low ODs, respectively (**Figure 1A**). The assay also showed a high correlation between ODs and sample dilutions when F peptide was used, with an R^2^ of 0.9773 (p < 0.0001), 0.9784 (p < 0.0001), and 0.9846 (p < 0.0001) for samples with known high, medium, and low ODs (**Figure 1A**).

Thereafter, OD concordance between MCPyV VP1 S and VP2 F peptides for the whole set of ES sera (n = 226) was evaluated by Spearman’s correlation analysis. Results indicate a good degree of correlation between ODs for S and F peptides, with a Spearman’s coefficient r of 0.7991 (p < 0.0001) (**Figure 1B**).

### Detection of Serum IgG Antibodies Against Merkel Cell Polyomavirus by Indirect ELISA in Elderly Subjects

In order to assess the distribution of MCPyV infection in elders, sera from ES (n = 226) were tested by indirect ELISA for IgG Abs reactivity to MCPyV VP1 S and VP2 F peptides/mimotopes. A similar overall prevalence of 67.7% (153/226) and 71.2% (161/226) was obtained in sera from ES reacting to S and F peptides, respectively (p > 0.05) (**Table 1**).

**Table 1:** Seroprevalence of IgG antibodies reacting with Merkel cell polyomavirus VP1 S and VP2 F peptides in healthy elderly subjects.

Age years	Number of samples	Male (%)	Number of positive samples (%)		
			VP1 S	VP2 F	VP1 S+VP2 F
66–70	40	52.5	28 (70)	26 (65)	25 (62.5)
71–75	46	50	33 (71.7)	39 (84.8)	33 (71.7)
76–80	57	56.1	39 (68.4)	38 (66.7)	37 (64.9)
81–85	47	36.2	32 (68.1)	32 (68.1)	30 (63.8)
85–100	36	36.1	21 (58.3)	26 (72.2)	19 (52.8)
Total	226	46.9	153 (67.7)	161 (71.2)	144 (63.7)

Serum samples (n = 226) were from healthy elderly subjects. Statistical analyses were performed using the two-sided chi-square test. No statistical differences were detected among age-stratified groups (p > 0.05).

Moreover, with a few exceptions, sera testing negative for S peptide did not react to F peptide. In detail, 7.5% (17/226) of sera resulting negative for S peptide were positive for F peptide, while 3.9% (9/226) of sera were negative for F, while being positive for S peptide. The combined overall prevalence, for both S and F peptides, in ES sera resulted as 63.7% (144/226) (**Table 1**). Serum reactivity to MCPyV was then determined in age-stratified ES, and rates were compared. A total of 62.5% (25/40), 71.7% (33/46), 64.9% (37/57), 63.8% (30/47), and 52.8% (19/36) of serum samples were found to be positive for IgG Abs reacting to MCPyV in ES cohorts aged 66–70, 71–75, 76–80, 81–85, and 86–100 years, respectively (**Table 1**). No statistically significant differences in MCPyV seroreactivity were determined among groups (p > 0.05).

In order to evaluate an association between MCPyV infection and gender, MCPyV seroprevalence was investigated in gender-stratified ES. To this purpose, the presence of IgG Abs against MCPyV was determined in sera from male (n = 106) and female (n = 120) ES, and rates were compared. The prevalence of serum Abs reacting to MCPyV resulted as 67% (71/106) and 61% (73/120) in male and female groups, respectively, with no differences between the two gender groupings (p > 0.05).

### Serological Profiles of IgG Reacting to Merkel Cell Polyomavirus in Elderly Subjects

**Figure 2:**
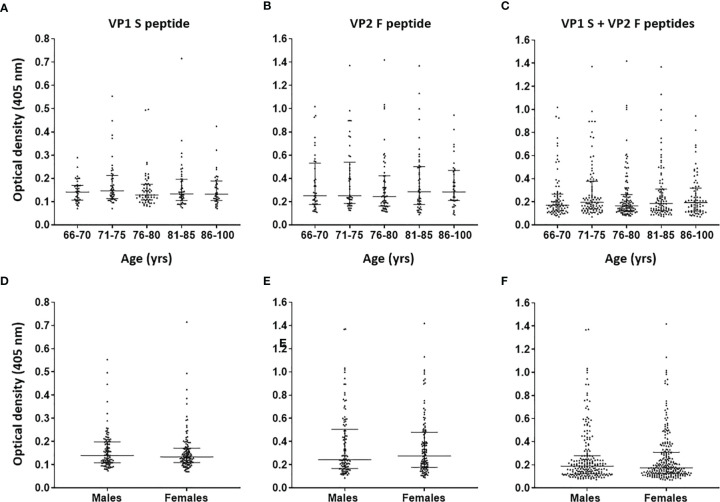
Serological profiles of serum antibody reactivity to Merkel cell polyomavirus (MCPyV) VP1 S **(A, D)**, VP2 F **(B, E)** peptides and for combined S and F peptides **(C, F)** in age- and gender-stratified elderly subjects (ES). Immunologic data are from age- and gender-stratified ES, and results are reported as optical density (OD) value readings at λ 405 nm for serum samples assayed in indirect ELISA. In the scatter dot plot, each dot represents the dispersion of ODs for each sample. The median is indicated by the line inside the scatter plot with the interquartile range (IQR) in age-stratified ES, i.e., 66–70 (n = 40), 71–75 (n = 46), 76–80 (n = 57), 81–85 (n = 47), and 86–100 (n = 36) years and in gender-stratified ES, i.e., males (n = 106) and females (n = 120). No statistically significant differences in MCPyV seroreactivity were determined among age-/gender-stratified groups (p > 0.05).

Serological profiles of IgG Abs reactivity to S and F peptides, both for single peptides and in combination, were analyzed. Immunological data were taken from the entire ES cohort (n = 226), and results are reported as OD readings at λ 405 nm. The median [interquartile range (IQR)] OD values for S peptide and F peptide in ES sera were 0.14 (0.10–0.18) and 0.25 (0.18–0.49), respectively, while for combined S and F peptides, these resulted as 0.18 (0.13–0.30). The median (IQR) ODs for S peptide and F peptide, both for single mimotopes and in combination, were then determined in age-stratified ES, and values were compared. The median (IQR) OD values for S peptide resulted as 0.14 (0.10–0.17), 0.15 (0.11–0.21), 0.13 (0.11–0.17), 0.13 (0.11–0.20), and 0.13 OD (0.10–0.19) in age-stratified ES groups aged 66–70, 71–75, 76–80, 81–85, 86–100 years, respectively (**Figure 2A**). The median (IQR) OD values for F peptide resulted as 0.25 (0.17–0.53), 0.25 (0.18–0.53), 0.24 (0.16–0.42), 0.29 (0.18–0.50), and 0.28 (0.20–0.46) in age-stratified ES groups aged 66–70, 71–75, 76–80, 81–85, and 86–100 years, respectively (**Figure 2B**). The median (IQR) ODs for combined S and F peptides resulted as 0.17 (0.12–0.26), 0.19 (0.13–0.37), 0.16 (0.12–0.26), 0.19 (0.12–0.31), and 0.19 (0.12–0.32) in age-stratified ES groups aged 66–70, 71–75, 76–80, 81–85, and 86–100 years, respectively (**Figure 2C**). No statistically significant differences in OD values for peptides S and F, both for single peptides and in combination, were determined among age-stratified ES groups (p > 0.05). Similarly, Spearman’s correlation analysis showed a lack of correlation between age and OD levels, for peptides S and F, in the entire cohort of ES (p > 0.05).

The median (IQR) ODs for S peptide and F peptide, both for single peptides and in combination, were then determined in ES males (n = 106) and females (n = 120), whereas values were compared. The ODs for S and F peptides in the male group were 0.14 (0.11–0.2) and 0.24 (0.17–0.50), respectively, while these resulted as 0.13 (0.11–0.17) and 0.28 (0.18–0.49), respectively, in the female group (**Figures 2D, E**). Moreover, the median (IQR) ODs for combined S and F peptides were 0.19 (0.12–0.28) in males and 0.17 (0.13–0.30) in females (**Figure 2F**). No statistically significant differences in ODs for peptides S and F, both for single peptides and in combination, were determined between males and females (p > 0.05).

### Serum IgG Reactivity to Merkel Cell Polyomavirus in Senior Elderly Subjects Versus Younger Healthy Subjects

**Figure 3:**
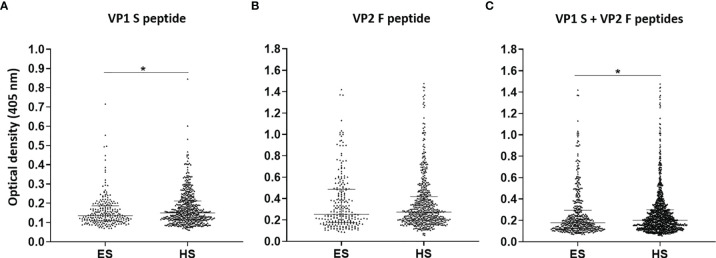
Serologic profiles of serum antibody reactivity to Merkel cell polyomavirus (MCPyV) VP1 S **(A)** and VP2 F **(B)** peptides and for combined S and F peptides **(C)** evaluated in elderly subjects (ES) compared with healthy subjects (HS). Immunologic data are from ES (n = 226) and HS (n = 548), and results are reported as optical density (OD) value readings at λ 405 nm for sera analyzed in indirect ELISA. In the scatter dot plot, each dot represents the dispersion of ODs for each sample. The median is indicated by the line inside the scatter plot with the interquartile range (IQR) in ES and HS cohorts. **(A)** *p < 0.05; **(C)** *p < 0.05.

In the second phase, MCPyV seroprevalence and serologic profiles in ES were compared with those obtained in sera from HS, which had previously been investigated in our laboratory (18). The prevalence of serum anti-MCPyV IgGs, combining S and F peptide reactivity, in HS was 62.9% (345/548) (18). No statistical differences in MCPyV seroprevalence were found either between the two entire cohorts of ES and HS (p > 0.05) or in MCPyV seroprevalence and serologic profiles among age-stratified ES and HS (p > 0.05). Notably, serologic profile analysis indicated that S peptide ODs resulted as lower in the ES group (n = 226, median [IQR], 0.13, 0.10–0.19) than in HS (n = 548, 0.15, 0.11–0.21, p < 0.05) (**Figure 3A**). Contrariwise, F peptide ODs were similar in ES (0.25, 0.18–0.49) and HS groups (0.27, 0.2–0.42) (p > 0.05) (**Figure 3B**). Lower ODs for combined S and F peptides were detected in ES group (0.18, 0.13–0.29) compared with HS (0.2, 0.14–0.3) (p < 0.05) (**Figure 3C**).

**Figure 4:**
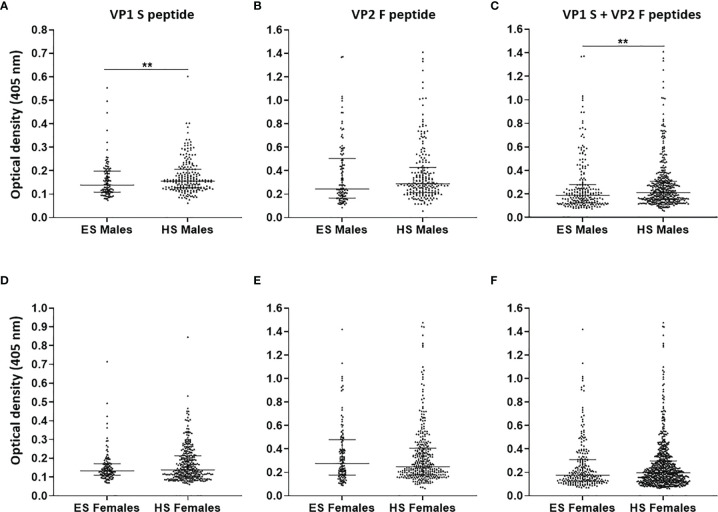
Serologic profiles of serum antibody reactivity to Merkel cell polyomavirus (MCPyV) VP1 S **(A, D)** and VP2 F **(B, E)** peptides and for combined S and F peptides **(C, F)** evaluated in gender-stratified elderly subjects (ES) compared with gender-stratified healthy subjects (HS). Immunologic data are from gender-stratified ES (n = 106 males and n = 120 females) and HS (n = 210 males and n = 338 females). Results are expressed as optical density (OD) value readings at λ 405 nm for serum samples assayed in indirect ELISA. In the scatter dot plot, each dot represents the dispersion of ODs for each sample. The median is indicated by the line inside the scatter plot with the interquartile range (IQR) for each group of subjects analyzed. **(A)** **p < 0.01; **(C)** **p < 0.01.

Afterwards, MCPyV seroprevalence was compared in ES (n = 106) and HS (n = 210) males, as well as in ES (n = 120) and HS (n = 338) females (18). A total of 65.2% (137/210) HS males and 61.5% (208/338) HS females presented IgGs against MCPyV (18). No statistically significant differences were determined among groups (p > 0.05). As regards the serologic profiles, ODs for S peptide resulted as lower in ES males (0.14, 0.11–0.2) than in HS males (0.16, 0.13–0.21, p < 0.01) (**Figure 4A**), while no statistical differences in F peptide ODs were determined in ES (0.24, 0.16–0.5) and HS males (0.29, 0.21–0.43) (p > 0.05) (**Figure 4B**). Furthermore, lower ODs for combined S and F peptides were detected in ES males (0.19, 0.12–0.28) when compared with HS males (0.21, 0.15–0.31, p < 0.01) (**Figure 4C**). The ODs for S peptide in ES females (0.13, 0.11–0.17) were similar when compared with those in HS females (0.14, 0.1–0.21, p > 0.05) (**Figure 4D**). Similar ODs for F peptide were also found in ES (0.28, 0.18–0.48) and HS females (0.25, 0.18–0.41, p > 0.05) (**Figure 4E**). No differences in ODs for combined S and F peptides were detected in ES (0.17, 0.13–0.31) and HS females (0.2, 0.13–0.3, p > 0.05) (**Figure 4F**).

## Discussion

Detailed information on MCPyV serology in the healthy elderly population is still being debated. Herein, the impact of MCPyV infection in ES was determined using an innovative indirect ELISA with two linear synthetic peptides that mimic MCPyV VP antigens (18). MCPyV seroprevalence and serologic profile in ES according to age and gender were therefore determined.

In a first phase, the performance of the indirect ELISA was evaluated by i) testing assay dilutional linearity (53, 59–61), or accuracy, for MCPyV VP1 S and VP2 F peptides and by ii) comparing S and F peptide ODs. Linear regression analyses showed a remarkable goodness of fit between sample dilutions and ODs, with an R^2^ ranging from 0.09768 to 0.09959 (p < 0.0001) being found, for both peptides. Furthermore, Spearman’s analysis, indicated good concordance between S and F peptide ODs, with an r of 0.7991 (p < 0.0001). These data indicate that not only is this assay robust in detecting anti-MCPyV IgGs with high accuracy, a key prerequisite for reliable ELISAs (18, 62–66), but also S and F peptides can be exploited simultaneously, showing high concordance. These data indicate that this method is accurate, while providing high reliability in detecting anti-MCPyV IgGs (18, 67, 68).

Analysis was afterwards carried out on sera from ES aged from 66 to 100 years with unknown MCPyV serology. Sera were investigated for IgG reactivity to MCPyV antigens. Sera were considered MCPyV-positive when IgGs reacted to both S and F peptides (18).

The overall prevalence, obtained by combining MCPyV-positive ES sera, for both peptides, resulted as 63.7%, a proportion similar to 62.9%, which we previously obtained in HS aged 18–65 years (18). These data suggest that MCPyV circulates asymptomatically not only in the adult population but also in elders at a relatively high prevalence. Over the years, serological evidence of circulating MCPyV in adults has been described with highly variating rates ranging between 46% and 87%, according to the study considered (3, 12–16, 19–26). Distinct prevalence figures across studies have been described for individuals aged over 60 years, with rates grouping from about 58%–70% (13, 14, 21–23, 29) to approximately 73%–95% (12, 15, 25–28, 69). Differences in geographical region/study populations could reflect these variations across studies (25, 69). However, variating rates have been reported across studies conducted in the same geographical region (15, 20, 22, 23, 25, 28, 29, 69, 70). Therefore, the current data available do not allow to reach robust conclusions regarding the presence of an MCPyV seroprevalence pattern according to the geographical region. Multicenter studies based on large sample sizes should be performed to clarify this issue. Moreover, methodological differences in immunoassays, which may result in variating rates, could also be considered an explanation for these variations across studies (21, 25). It should be recalled that previous MCPyV-based immunological methods mainly employ VLPs as antigens for immunoreactions, which are *in vitro*-generated recombinant MCPyV VPs (3, 12, 14, 20, 27, 71, 72). VLP-based assays might be susceptible to overestimation/cross-reaction, as VLPs share common antigens with other PyVs (16, 27, 73), therefore potentially hampering the result (27, 73). Our immunoassay differs from previous methods since it is based on two synthetic linear peptides mimicking portions of MCPyV VPs. Indeed, the S and F peptide a.a. chains used herein present a very marginal identity with corresponding VPs from other PyVs, as has been demonstrated (18). In addition, VLP-based systems necessitate time-consuming/expensive protocols (27, 73), which might be susceptible to methodological flaws. Considering these aspects, our indirect ELISA exhibits high specificity in detecting anti-MCPyV IgGs, while cost/time efficiency and a simple procedure are also provided (18).

In terms of MCPyV seroprevalence, no variations with age were determined in ES (p > 0.05). Comparable rates were also obtained when comparing age-stratified ES cohorts with age-stratified HS cohorts from our previous study (p > 0.05) (18). This result suggests that MCPyV serology appears to not differ from adulthood to middle/advanced age, persisting lifelong without significant fluctuations. Despite previous studies being characterized by variating MCPyV seroprevalence rates (74), our results are in agreement with previous studies reporting a lack of/slight variations with age (12, 14, 21, 22, 24, 26, 27, 69). MCPyV establishes a lifelong asymptomatic infection in adults. In physiological conditions, this kind of infection may provide a long-lasting/lifetime source of viral antigens, which are permanently exposed to the host immune system, while continuous, consequent antigen stimulation is provoked (14, 24). An alternative scenario provides for the gradual production/exposure of MCPyV antigens to the immune system during adulthood, which leads to a gradual immune response increase in an age-dependent manner (75), as described for other viruses (76). As for other PyVs (33), the gradual production of antiviral IgGs might be prompted by an increase in MCPyV replication levels or reinfections with different strains throughout life (21, 25). Consistently, MCPyV seroreactivity has also been reported to increase with age (13, 23, 28, 70).

It is important to point out that lower OD levels were found in ES compared with HS from our previous study (p < 0.05) (18). In addition, despite the entire ES group having statistically similar MCPyV seroprevalence rates, the older cohort, aged 86–100 years, exhibited the relatively lowest rate, i.e., 53%, between 10% and 20% lower than the remaining cohorts aged below 85 years. These results, in agreement with previous findings, suggest a possible decrease in IgG levels against MCPyV during senility (15, 25, 26). Indeed, the immune system is prone to decline in old-aged subjects (77), potentially leading to a reduced response to MCPyV. At the same time, as high MCPyV DNA amounts have been described in elders, a link between age and an increase in viral replication levels has also been theorized (34). Physiological immune senescence could therefore favor higher levels of MCPyV replication, as a minor response to MCPyV may occur (29, 32, 34, 78), which in turn might lead to MCC carcinogenesis. This is understandable, as MCC mainly arises during advanced senility following MCPyV reactivation (35, 36). The impairment of antiviral/cancer immune surveillance in advanced age can therefore favor MCPyV-driven MCC carcinogenesis (38, 47). However, Spearman’s analysis and comparisons among, and within, age-stratified ES and HS cohorts showed a lack of OD variation with age. The relatively small fraction of serum samples available for immunoreactions characterizing the age-stratified ES cohorts could justify this result. Further investigations with larger study cohorts might clarify the presence of variations in MCPyV seroreactivity according to age.

In summary, the data in the present study, along with evidence reported previously (12, 14, 21, 22, 24, 26, 27, 69), cumulatively suggest that, after seroconversion early in life (22, 69), MCPyV infection seems to remain stable throughout adulthood by evoking a physiological immune response, which might be followed by a decrease in MCPyV seroreactivity during advanced senility. The decrease in antiviral/cancer immune surveillance might lead to higher MCPyV replication levels ultimately leading to MCC onset (38, 47).

No differences in terms of both MCPyV seroprevalence and ODs were determined between ES males and females (p > 0.05). These data, in agreement with those reported previously (18, 25–28, 69), support the view that MCPyV infection is homogenously distributed in humans without gender-based disparity. Notably, a decrease in ODs with age was observed in males, but not in females, with low levels being found in ES males compared with HS males from our previous study (p < 0.05) (18). This evidence suggests that males might be more predisposed to develop immune senescence than females, which in turn might possibly favor higher MCPyV replication levels. A gender dimorphism in immune response impairment with age has been described as more pronounced in males than females (79). Indeed, although some evidences indicate a lack of gender differences (80), MCC seems to occur more frequently in males than females (81–84).

As MCPyV LT/sT plays a role in MCC onset/development (7–9), investigating the presence of serum IgGs against these viral oncoproteins in the healthy population, to identify MCC-risk individuals, might present clinical relevance. Our ELISA method can potentially be extended by using different linear peptides/mimotopes mimicking portions of MCPyV LT/sT antigens, as performed previously (85). This methodological approach, which might allow a more comprehensive evaluation of the MCPyV infection in the healthy population, in immunosuppressed individuals and in MCC-risk patients, will be employed in further studies.

Our study presents some limitations. First, S and F peptides, used herein as mimotopes to detect circulating IgGs against MCPyV, were not tested for the detection of IgGs against MCPyV variants with mutations in the sequences corresponding to the used peptides. However, *in silico* analyses conducted previously to assess the theoretical reliability of the two peptides as mimotopes/antigens indicated that both peptides were able to detect IgGs to a variety of known MCPyV strains with high concordance (18). Second, a relatively small number of sera from ES have been tested. However, the sample size employed herein is statistically appropriate and significant.

In conclusion, evaluating the impact of MCPyV infection in ES is of paramount importance for identifying individuals potentially at risk of MCC. Our indirect ELISA proved to be reliable in investigating IgGs reacting to MCPyV VP mimotopes in ES sera. Circulating anti-MCPyV IgGs were determined in sera from ES, while a lack of age-/gender-related variations in terms of MCPyV seroprevalence and serologic profile was found. The results of the present study suggest that oncogenic MCPyV circulates in the elderly asymptomatically without variations according to age and gender, at a relatively high prevalence. However, the entire ES group reported lower ODs compared with HS, while lower ODs were also found in ES males compared with HS males. This result may suggest that immunesenescence might be responsible for a decreased IgG antibody response to MCPyV, thereby potentially leading to an increase in MCPyV replication levels. In a few cases, alongside other risk factors, this phenomenon might prompt MCPyV-driven MCC onset, as described in elders aged over 60 years.

## Data Availability Statement

The raw data supporting the conclusions of this article will be made available by the authors, without undue reservation.

## Ethics Statement

The studies involving human participants were reviewed and approved by County Ethical Committee, Ferrara, Italy (ID:151078). The patients/participants provided their written informed consent to participate in this study.

## Author Contributions

Conceptualization: MT and JR. Methodology: CM and JR. Software: CM and JR. Formal analysis: CM. Investigation: CM and GP. Resources: MG. Data curation and statistical analysis: CM. Writing—original draft preparation: CM and JR. Writing—review and editing: MT, FM, and MG. Visualization: CM, CL, and EM. Supervision: JR and MT. Funding acquisition: JR and MT. All authors contributed to the article and approved the submitted version.

## Funding

This work was supported, in part, by grants IG 21956 (to JR), IG 21617 (to MT) from the Associazione Italiana per la Ricerca sul Cancro (AIRC), Milan, Italy, and University of Ferrara, FAR grants 2021 (FM and MT).

## Conflict of Interest

Authors CM, CL, EM, FM, MT, and JR are holders of the Italian patent application number 102020000021235 (I0188839) BRE-mma, filed on September 8, 2020. Data of this work were enclosed, in part, in the aforementioned Italian patent.

The remaining authors declare that the research was conducted in the absence of any commercial or financial relationships that could be construed as a potential conflict of interest.

## Publisher’s Note

All claims expressed in this article are solely those of the authors and do not necessarily represent those of their affiliated organizations, or those of the publisher, the editors and the reviewers. Any product that may be evaluated in this article, or claim that may be made by its manufacturer, is not guaranteed or endorsed by the publisher.
